# Irreparable Cuff Injuries: Treatment Options and Clinical Outcomes

**DOI:** 10.1055/s-0044-1788784

**Published:** 2024-09-04

**Authors:** José Carlos Souza Vilela, João Felipe de Medeiros Filho, Tadeu Fonseca Barbosa, Lucas de Castro Melo Deligne, Thalles Leandro de Abreu Machado

**Affiliations:** 1 Grupo de Cirurgia do Ombro, Hospital Unimed BH, Belo Horizonte, MG, Brasil; 2 Grupo de Ombro, Hospital Mater Dei, Belo Horizonte, MG, Brasil; 3 Universidade Federal do Rio Grande do Norte, Natal, RN, Brasil

**Keywords:** outcome treatment, rotator cuff tear, shoulder

## Abstract

Rotator cuff tear is a common source of pain and disfunction in shoulder, with prevalence increasing with age. Nonsurgical treatment is adequate for many patients; however, for those for whom surgical treatment is indicated, rotator cuff repair provides reliable pain relief and good functional results. However, massive and irreparable tears due to tear size, tendon retraction, muscle atrophy, fatty infiltration are a significant challenge for surgeons. Whenever irreparable tears coexist with cartilage degeneration/arthritis (rotator cuff arthropathy), the indication of reverse shoulder arthroplasty is the golden standard. However, in young patients without arthritis, joint preserving procedures, from debridement to muscle transfers, are preferred. Choosing the most appropriate treatment is not quite established in literature, each treatment modality present particular indications, advantages and disadvantages.

## Introduction

The first mention of irreparable cuff injury in the literature might be from Wiley who, in 1991, 1 despite not providing a precise concept or adequate treatment, stated that: "large injuries may be irreparable surgically and under such circumstances, debridement of the cuff edges and bursa decompression can alleviate pain”. Even today, the concept is not precise, and the treatment is not assertive. The concepts of irreparable injury, extensive injury, and tendon healing are confusing and, ultimately, determine the clinical outcome.

This paper does not aim to define the precise concepts of an extensive injury and an irreparable injury since they are not clear in the literature. Similarly, there is no definition of healing failure (pain, reoperation, decreased strength, etc.).

An extensive injury presents two or more ruptured tendons, a lesion over 5 cm in length, or both.

The concept of irreparability is more imprecise because it implies the impossibility of pulling the tendon and fixating it at its anatomical attachment site, or in a medialized position, immediately lateral to the articular cartilage, with no tension. However, in concrete terms, this perception only occurs perioperatively. The following parameters routinely infer this irreparability:

- an acromiohumeral height < 6 mm due to the cuff's lack of opposition to the deltoid muscle traction.
- torn edge of the tendon retracted at the level of the glenoid joint line (Pate III).
- significant muscular atrophy at the belly of the supraspinatus muscle (positive tangent sign).
- significant fatty infiltration at the belly of the affected muscle (Goutallier >2). 2

Some patient-related factors negatively influence healing and deserve consideration, including age > 70, smoking, diabetes, hypercholesterolemia, multiple previous steroid infiltrations, cooperation and adherence to treatment, and secondary gains. 2 3

In summary, preoperative factors indicating low or no possibility of functional repair are lesion size greater than 5 cm, fat replacement of the affected belly > 50% (Goutallier III or more), muscle atrophy > 50% at the supraspinatus muscle belly (tangent sign on magnetic resonance imaging in sagittal sequence), retraction of the ruptured tendon stump at the glenoid level (Patte III) and fixed superior subluxation of the humeral head, with acromiohumeral height < 6 mm (Hamada 2 or more). We believe these patients have irreparable injuries and present the existing treatment options in the next section. 2 3

## Treatments

- Clinical Treatments
- Surgical Treatments sparing the joint
- Surgical Treatments replacing the joint

## Clinical Treatment

Non-surgical treatment is INITIALLY indicated for all symptomatic patients and continued for patients with low functional demand and comorbidities contraindicating surgical treatment. It encompasses physiotherapeutic rehabilitation focused on physical anti-inflammatory and analgesic measures, strengthening the remaining cuff, and strengthening the parascapular and deltoid muscles. The use of non-steroidal anti-inflammatory medications and corticosteroid infiltrations should occur sparingly. There is no scientific evidence to warrant using hyaluronic acid or platelet-rich plasma (PRP) injections. 2

## Debridement, Acromioplasty, and Tenotomy with or without Tenodesis

All these treatment modalities, combined or alone, resulted in immediate clinical improvement in around 95% of patients but with progressive deterioration in pain and function. Walch et al. 4 presented the first systematic multicenter report involving 283 patients with a mean age of 64.3 years and a mean follow-up period of 57 months. These authors observed that the initial gain was very satisfactory in 93% of patients. However, there was a decline in satisfaction and function in subsequent years, resulting in loss of external rotation, atrophy, weakness, and progression of cartilage degeneration. They also noted that tenotomy has an adverse effect on patients with an acromiohumeral distance > 7 mm, and that involvement of the teres minor has a poor prognostic value.

Pander et al. 5 retrospectively evaluated 39 patients with a mean age of 75.6 years who underwent debridement of irreparable injuries with or without tenotomy and a mean follow-up period of 6.5 years. Despite some limitations, like not including patients requiring an additional surgical procedure due to an unsatisfactory clinical outcome, these authors observed that it is possible to obtain favorable outcomes in the elderly population with low functional demand.

Boileau et al. 6 observed that biceps tenotomy promoted an average of 1.1 mm of head elevation but is not a risk factor for progression to rotator cuff arthropathy. These authors suggested that pseudoparalysis and rotator cuff arthropathy are contraindications for this procedure.

Checchia et al. 7 also demonstrated in 12 patients undergoing tenotomy, with an average follow-up period of 26 months, an improvement in satisfaction, range of motion (ROM; 30° of elevation, 1.7° of lateral rotation, and two vertebral levels of internal rotation). In their series, a single subject presented a characteristic Popeye deformity. 7

Almeida et al. showed that only 35.1% of patients undergoing tenotomy complained of biceps deformity. These authors reported that age affected the side and biotype had no significant correlation with deformity complaints. In contrast, the male gender, dominant side, abdominal skinfold < 23.2, triceps skinfold < 14.5, and body mass index (BMI)< 30 were risk factors for deformity. 8

In summary, it is better to reserve debridement, acromioplasty, and biceps tenotomy, whether or not associated with tenodesis, for elderly patients with low functional demands. As advantages, these are quick, simple techniques with few complications compared with other surgical procedures. More than a third (35.1%) of patients undergoing tenotomy complain of residual deformity (Popeye sign). These procedures, combined or alone, show immediate clinical improvement in 93% of patients, which deteriorates over time. Involvement of the teres minor muscle tendon, static superior subluxation with acromiohumeral distance < 6 mm, cartilaginous degeneration, and pseudoparalysis are contraindications.

## Partial Repair and Margin Convergence

Burkhart was the first author to introduce the concepts of functional rotator cuff tear and partial repair. This implies that the “ties” or margins of the lesions can keep the cuff functional despite the central lesion as long as these residual forces are balanced/compensated (also known as “even forces”). This is the logical reasoning for partial repair, which creates a balanced moment of force in the shoulder. Often, this scenario is the repair of an acute over a chronic injury, when the chronic portion of the injury is irreparable due to poor tendon quality and great retraction, and the acute part is repairable. When the acute portion heals, it is possible to resume the pre-traumatic state of balance, and the cuff is functional because of the restoration of even strength. Despite being small, Burkhart's initial sample had 14 subjects (average age, 56 years; average follow-up period, 20 months) and showed important gains in ROM, strength, and patient satisfaction. 9

In addition to partial repair, another surgical resource to biomechanically increase the repair of extensive injuries is margin convergence. After the partial repair, when the anterior and posterior edges of the cuff reach the footprint with acceptable tension and the central part does not, usually in “V" or “U” shaped injuries, the surgeon brings together the anterior and posterior edges and suture them side-to-side. This results in a significant reduction in the cuff pullout force (a six-fold reduction), minimizing fixation failure and improving clinical outcomes, which remain stable in the medium term. 10 11

We concluded that the indication for partial repair, with or without margin convergence, is appropriate for extensive acute over chronic injuries (traumatic cases) sparing a portion of the subscapularis tendon. The best outcomes occur after repairing the subscapularis and infraspinatus tendons, restoring the force balance, and making the cuff functional, even with the persistent injury in its central part. In the medium term, 67% of patients present good and excellent outcomes. These outcomes are better than isolated debridement. 8 9 11

## Subacromial Balloon

The subacromial balloon/spacer is an inflatable device made from poly-DL-lactide and E-coprolactone that degrades within 12 months. The procedure is simple and involves placing the device between the humeral head and the acromion, either by arthroscopy or percutaneously. It aims to restore the painless ROM of the shoulder in the presence of an irreparable injury by reducing subacromial friction and improving the deltoid lever by lowering the humeral head.

Senekovic was one of the first to demonstrate good outcomes with this technique. He showed that in 24 patients followed up for 5 years, i.e., after complete balloon reabsorption, 84.6% showed improvement, and only 10% presented worsening. The procedure is simple and the average time for device placement is 4.3 minutes. 12

The literature indicates an improvement in pain and functional disability in 46% to 84.6% of cases and a complication rate of 16.7%. The most frequent complications include anterior migration of the balloon, transient deficit of the lateral cutaneous nerve of the forearm, and infection. Irreparable damage to the subscapularis is a contraindication due to the risk of anterior extravasation. 13

**Fig. 1:**
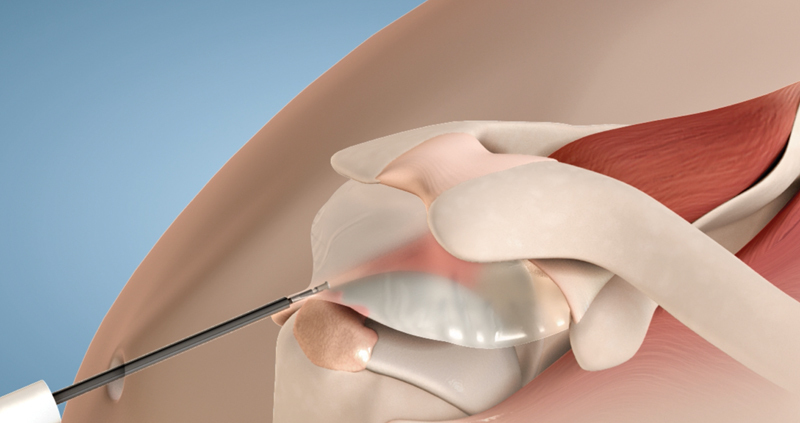
Schematic drawing of the subacromial balloon.

This procedure is suitable for patients with irreparable damage to the integrity of the subscapularis and teres minor, no osteoarthritis, and preferably with low demand. Another less classic indication is protecting the suture of a cuff injury. ( Fig. 1 ). 12 13

## Superior Capsule Reconstruction (SCR)

Mihata et al. 14 developed the concept of implanting the fascia lata in the glenoid and greater tubercle to stabilize the humeral head and prevent its upward ascension characterizing irreparable injuries to restore the rotation center of the humeral head. This concept was tested in vitro in multiple biomechanical studies and has been clinically reproduced since then. 14 Humeral head fixation to the glenoid with a fascia lata graft presents better resistance than the interposition of the tendon stump with other grafts. The most frequently interposed grafts are the fascia lata graft (autologous or homologous), acellular dermal graft, or the biceps tendon (long portion). The optimal procedure is to suture the remaining edges of the cuff to the graft (partial repair). 15

The literature reports diverse clinical outcomes because there is a lot of heterogeneity between surgical procedures: number of fascia lata layers, graft types (fascia lata, biceps, etc.), fixation site method, and surgeon experience. While some authors do not present consistently good outcomes, Mihata described 31 shoulders from 23 patients with a mean age of 65.1 years presenting significant improvement of 64° in anterior elevation, 14° in external rotation, and two vertebral levels of internal rotation. 14 15 16

Denard et al. 17 used dermal grafts and reported a surgical review rate of 18.6% in 59 patients. Despite observing complete healing in only 45% of patients, the procedure was successful in 74.6% of subjects.

This information warrants SCR indication for patients with irreparable cuff injuries, no cartilaginous degeneration, refractory to clinical treatment, intact or repairable subscapularis, and Hamada 1 or 2.

## Tendon Transfer

The classic indication for tendon transfer is young patients presenting a relative contraindication for reverse arthroplasty, no cartilaginous degeneration, and irreparable damage to the rotator cuff. Tendon transfer can provide satisfactory and long-lasting functional outcomes in this subset of patients. Currently, arthroscopy can assist most transpositions. As a general rule, the transposed muscle provides force at a lower level than the native muscle under physiological conditions. 18 19

When considering tendon transposition, there are important principles to follow:

1 - The transferred muscle must be expendable, with no compromise to the function of the donor limb.
2 - The donor and recipient muscles must have similar excursion and tension.
3 - The tension vector of the donor and recipient muscles must be similar.
4 - The transferred muscle must replace a function of the recipient's muscle. 18 19

## Posterosuperior Injuries

Historically, the latissimus dorsi has been the muscle of choice for this transfer. However, recently, the lower trapezius has grown in popularity due to ease, clinical outcome, and adherence to transfer principles.

## Latissimus Dorsi

It is a large muscle acting in arm adduction, extension, and medial rotation. It originates from the thoracolumbar fascia, spinous processes from T2 to L5, the dorsal surface of the sacrum and iliac crest, and muscular fascicles from three or four lower ribs interdigitating with the external abdominal oblique muscle. Its attachment is a terminal tendon 7 to 10 cm long and 0.5 to 1.5 cm wide after axial torsion of 180° at the bottom of the bicipital canal of the humerus, between the pectoralis major anteriorly and the teres major posteriorly. The thoracodorsal nerve, a branch of the posterior cord of the brachial plexus, provides its motor innervation. Its primary vascularization comes from the thoracodorsal artery, a terminal branch of the subscapular artery together with the circumflex artery of the scapula. From an anatomical point of view, two structures are at risk when transferring this muscle: the axillary nerve, which is close to its attachment site and in the transposition path, and the radial nerve, which is medial to its attachment.

Careful patient selection is crucial to obtaining satisfactory and lasting outcomes. Indications for transposition include extensive lesions in young, active patients with no cartilaginous degeneration. Risk factors for poor prognosis, therefore contraindications, are irreparable injuries to the subscapularis tendon, limitation of passive movement, pseudoparalysis, osteoarthritis, Hamada 3 or more, and fatty infiltration at the teres minor. 18 19

Gerber popularized the technique and showed excellent long-term outcomes, with 74% transfer viability and good and excellent outcomes after 10 years. The Subjective Shoulder Value (SSV) score increased from 29% to 70%, with a significant increase in ROM and strength, and failure in 10% of cases. 20

The attachment site is a source of controversy. Some believe that when it is more anterior (supraspinatus attachment), it increases the tenodesis effect; when it is more posterior (infraspinatus attachment), it produces better moments of force for abduction and external rotation. 18

Simultaneous teres major and latissimus dorsi transposition did not significantly increase the clinical outcome, not warranting its recommendation.

The current technical and material improvements allowed fully arthroscopic or assisted transpositions presenting the same clinical outcomes and complications. 18

## Trapézio Inferior

O músculo trapézio possui três partes: superior, média e inferior que funcionam juntos para elevar, retrair e rodar lateralmente a escápula. Ele se origina do osso occipital e dos processos espinhosos de C7-T12. A porção superior se insere no terço lateral da clavícula e e a média e inferior na parte medial do acrômio e espinha da escápula. Ele recebe sua irrigação da artéria cervical transversa e inervação do nervo acessório espinhal (XI nervo cranial).

O trapézio inferior é uma alternativa mais anatômica quando comparado com o latíssimo do dorso. Ele tem origem mais cranial que o latíssimo do dorso e mais medial que a fossa do infraespinal, mantendo exatamente o mesmo vetor de força. Dessa forma, propicia melhor momento de força em rotação externa. Uma ressalva dessa técnica descrita por Elhassan é que o comprimento do tendão é insuficiente para se alcançar o grande tubérculo, necessitando dessa forma de um enxerto autólogo ou homólogo que pode ser dos flexores do joelho ou aquiles.

Essa transferência propicia uma melhora/aumento da rotação externa de 70°, priorizando se dessa forma os pacientes que apresentam perda mais importante da rotação externa do que a elevação ( Fig. 2 ). 18 19

**Fig. 2:**
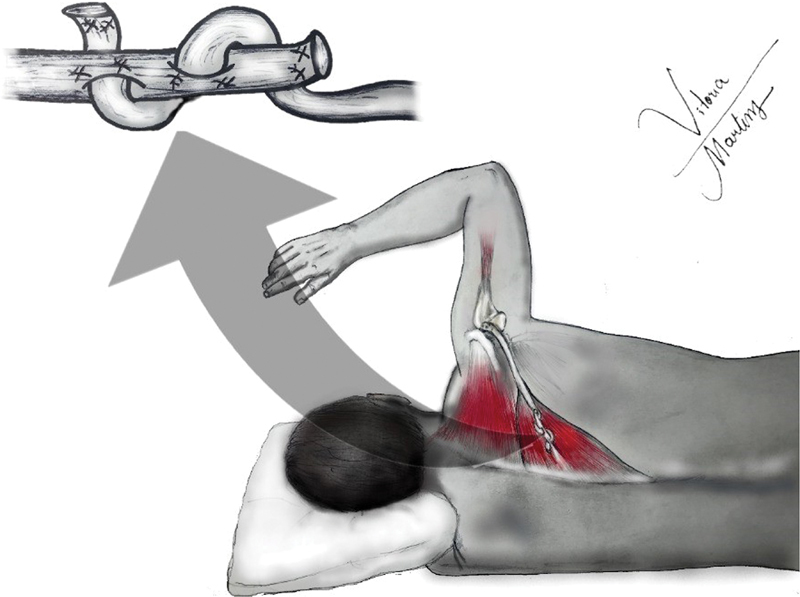
Ilustração da transferência do trapézio inferior.

## Lower Trapezius

The trapezius muscle has three portions, i.e., upper, middle, and lower. These parts work together to elevate, retract, and laterally rotate the scapula. The muscle originates from the occipital bone and spinous processes from C7-T12. The upper portion attaches to the lateral third of the clavicle and the middle and lower portions attach to the medial part of the acromion and scapular spine. The lower trapezius receives irrigation from the transverse cervical artery and innervation from the spinal accessory nerve (XI cranial nerve).

The lower trapezius is a more anatomical alternative than the latissimus dorsi. It has a more cranial origin than the latissimus dorsi and is more medial than the infraspinatus fossa, maintaining the same force vector. As such, it provides a better moment of force in external rotation. One caveat of this technique described by Elhassan is that the length of the tendon is insufficient to reach the greater tubercle, requiring an autologous or homologous graft, which may be from the knee flexor or calcaneus tendon.

This transfer provides an improvement/increase in external rotation of 70°, thus prioritizing patients presenting a more significant loss of external rotation than elevation. ( Fig. 2 ). 18 19

**Fig. 2:**
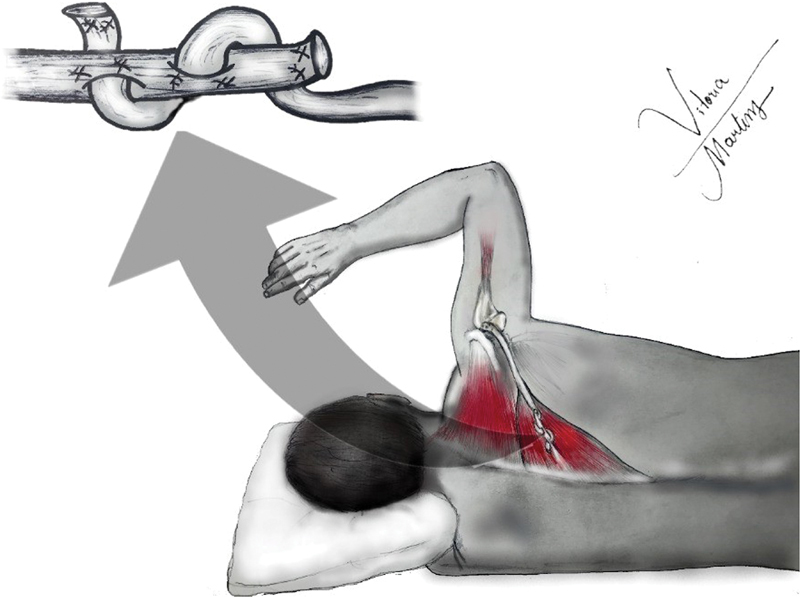
Illustration of lower trapezius transfer.

## Anterosuperior Injuries

### Pectoralis Major

The pectoralis major muscle adducts, flexes, and medially rotates the humerus. It has two portions, i.e., the clavicular and sternal portions. The clavicular portion originates from the medial aspect of the clavicle, and the sternal portion comes from the upper portion of the sternum and the second to fourth ribs. These portions attach to the lateral part of the intertubercular groove. Pectoralis major irrigation comes from the pectoral branch of the acromial thoracic trunk and the medial and lateral pectoral nerves provide its innervation.

Its transfer occurs most commonly for anterior superior injuries, to replace irreparable injuries to the subscapularis tendon. In its initial description by Wirth and Rockwood, the transposition was superficial to the conjoint tendon. Later, Resch revised the technique, and the transposition was deep to the conjoint tendon, increasing mechanical efficiency. Pay special attention to the musculocutaneous nerve during the deep transposition to the conjoint tendon.

Clinical outcomes are predictable for pain relief and heterogeneous concerning functional gain (ROM and strength). Outcomes are better when sparing the supraspinatus muscle tendon and centering the head. In contrast, cases with humeral head subluxation present worse functional outcomes. 18

A theoretical explanation for the non-reproducibility of the functional gain in transferring the pectoralis major is that in certain abduction positions, the force vector of the pectoralis is orthogonal to the vector of the subscapularis, even causing anterior subluxation of the humerus. 18 19

### Latissimus Dorsi

The approach to the latissimus dorsi occurs via the axillary fossa. Then, proceed to latissimus dorsi attachment, with or without the teres major, into the lesser tubercle, whether open or arthroscopically. This technique is less likely to cause iatrogenic nerve injury and better reproduces the subscapularis traction line. 15 16

### Reverse Prosthesis

The gold standard indication for reverse arthroplasty is rotator cuff arthropathy (RCA). This single procedure solves two issues: joint degeneration and rotator cuff insufficiency resulting from irreparable injury, the latter being difficult to treat with other surgical modalities.

While the indication for reverse prosthesis is well-established in RCA, it is not clear for irreparable injuries with no osteoarthritis. The indication must rely on individualized criteria. 21

The ideal candidate must be over 65 years old and present pain and pseudoparalysis in a scenario of irreparable injury, and an anterior elevation lower than 90°. Additionally, elderly patients with other poor prognostic factors such as smoking and diabetes may be eligible for reverse arthroplasty, as well as patients who, even with no osteoarthritis, present leakage/instability of the humeral head. The literature shows that patients undergoing reverse arthroplasty at an age < 65 years have worse clinical outcomes and higher complication rates. 22

Relative contraindications are patients younger than 65, with neurological dysfunction of the upper limb, and virtually normal function with an elevation higher than 90°, Simple Shoulder Test (SST) functional questionnaire score > = 7, or both, denoting satisfactory function. 22 Until recently, deltoid muscle dysfunction was also a contraindication. However, Elhassan et al. demonstrated good outcomes in reverse arthroplasties associated with pectoralis transposition in patients with deltoid paralysis. 23

Frankle et al. 24 evaluated 60 patients for an average follow-up period of 33 months and showed a significant improvement in anterior elevation (55° to 105°), abduction (41° to 102°), lateral rotation (12° to 41°) and visual analog scale (VAS) for pain (6.2 to 2.2). Regarding complications, they observed 17% of scapular notching and 12% of revision due to failure of the glenoid platform. Multiple studies by many surgeons reproduced these outcomes. More recent studies, such as those from Groh and Groh, showed lower complication (7%) and reoperation (5.3%) rates. 25

**Fig. 3:**
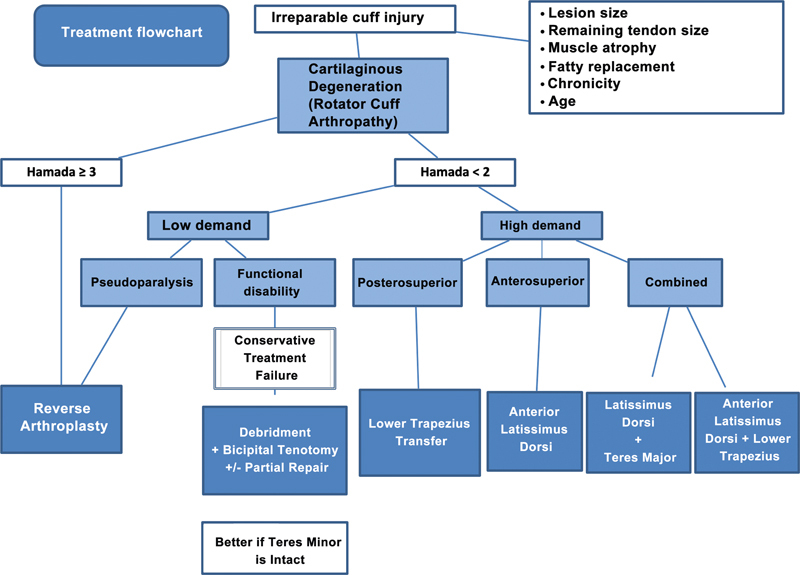
Flowchart for treating irreparable cuff injuries.

The biggest concern when placing a reverse prosthesis may be the prosthesis longevity, especially in younger patients. The literature shows 91% to 95% of prosthesis survival in 10 years. Unlike arthroplasty for other conditions, RCA and irreparable injuries show less functional deterioration after the fifth year of surgery ( Fig. 3 ). 26

## Final Considerations

Treatment of irreparable cuff tears in elderly patients with low functional demands and osteoarthritis (rotator cuff arthropathy) presents a predictably good outcome with the indication of a reverse prosthesis. However, there is a large subgroup of young and active patients who meet irreparability criteria. In this subgroup, despite the enormous advances in surgical treatment modalities in recent years, from partial repair using a subacromial balloon to superior capsule reconstruction and tendon transfers, the outcomes are less predictable despite the particularity of each patient and the injury (anterosuperior, posterosuperior, etc.), functional demand, adherence to treatment, comorbidities and, MAINLY, the experience of the attending physician with the respective techniques. 26

In the literature, most studies present level III and IV evidence. Due to the lack of adequate comparative studies, there is no consensus regarding the superiority of one of these techniques over the others. Therefore, treatment must be individualized, weighing up the advantages and disadvantages of each modality for the patient.

In summary, considering the best available evidence, **partial repair** is indicated in acute-on-chronic injuries, given the reparable nature of the acute portion of the injury, and in patients with no osteoarthritis and low functional demand, with outcomes similar to more invasive methods. The **subacromial balloon** is a new method, without approval from many regulatory agencies, and indicated for patients without osteoarthritis, with preserved ROM, and intact subscapularis. However, the subacromial balloon requires more robust studies to confirm its indication. **RSC** presents excellent outcomes in the hands of surgeons experienced in this technique. RSC may treat pseudoparalysis but leads to frustrating outcomes in irreparable subscapularis injuries. **Tendon transfers of the latissimus dorsi and lower trapezius** for posterosuperior injuries and of the pectoralis major or latissimus dorsi for anterosuperior injuries are good indications for young patients with significant functional demands and no osteoarthritis. Finally, the reverse prosthesis is an excellent indication for elderly patients with low functional demands, with or without osteoarthritis. In patients under 60, with high functional scores, great functional demand, neurological deficit, or any combination of these factors, clinical outcomes are worse.
