# Supplementary material for: Irreparable Cuff Injuries: Treatment Options and Clinical Outcomes
Source: Rev Bras Ortop (Sao Paulo). 2024 Sep 4;59(4):e489–96. doi: 10.1055/s-0044-1788784 (PMC11374407; doi:10.1055/s-0044-1788784)
Supplement: Related texts

# Research article: Lesões irreparáveis do manguito: Opções de tratamento e desfechos clínicos

**Keywords:** lesões do manguito rotador, ombro, resultado do tratamento

## Abstract

A lesão do manguito rotador é uma fonte frequente de dor e incapacidade funcional na população em geral, e sua prevalência aumenta com o envelhecimento. O tratamento não cirúrgico é adequado para muitos pacientes, e, quando necessário, o reparo cirúrgico resulta em alívio da dor e bons resultados funcionais. Entretanto, lesões extensas e não reparáveis devido ao tamanho da lesão, grau de retração, atrofia muscular e substituição gordurosa apresentam desafios ao tratamento. Nos casos de lesões irreparáveis que coexistem com degeneração articular/osteoartrite (artropatia do manguito rotador), a indicação de artroplastia reversa é o padrão ouro. Porém, nos casos de pacientes mais jovens e sem osteoartrite, as indicações que preservam a articulação, desde o debridamento até as transferências tendinosas, são preferíveis. A escolha do tratamento mais apropriado não está claramente estratificado na literatura e cada modalidade tem suas indicações, vantagens e desvantagens.

## Introdução

Possivelmente a primeira menção de lesão irreparável do manguito na literatura foi de Wiley em 1991, 1 que apesar de não fornecer um conceito preciso e nem o tratamento adequado, disse que: "lesões muito grandes podem ser irreparáveis cirurgicamente e sob tais circunstâncias, o debridamento das bordas do manguito e a descompressão da bursa podem aliviar a dor”. Ainda hoje, o conceito não é preciso e o tratamento não é assertivo. Confunde-se os conceitos de lesão irreparável, lesão extensa e cicatrização do tendão, que é em última análise é o que determina o desfecho clínico.

Não é o objetivo do presente artigo estabelecer os conceitos precisos do que é uma lesão extensa e o que é uma lesão irreparável, visto que mesmo na literatura isto não está claro. Da mesma forma, o que pode ser considerado falha da cicatrização (dor, reoperação, diminuição de força, etc.) também não está definido.

Considera se lesão extensa a que apresenta um número de dois ou mais tendões rompidos e/ou uma lesão maior que 5cm de extensão.

O conceito de irreparabilidade é mais impreciso porque subentende que o tendão não pode ser tracionado e fixado até seu local de inserção anatômico, ou medializado, imediatamente lateral a cartilagem articular, sem tensão. Porém, de maneira concreta, essa percepção só se dá no peroperatório. Entretanto, há parâmetros que são utilizados rotineiramente para inferir essa irreparabilidade, são eles:

- altura úmero acromial <6mm, devido a falta de oposição do manguito à tração do músculo deltoide.
- Borda rompida do tendão retraída à altura da linha articular da glenoide (Pate III).
- Considerável atrofia muscular do ventre do músculo supraespinhal (sinal da tangente positivo)
- Importante infiltração gordurosa do ventre do músculo acometido (Goutallier >2). 2

Por fim, há também fatores relacionados ao paciente que influenciam negativamente a cicatrização que também devem ser considerados: idade > 70 anos, tabagismo, diabetes, hipercolesterolemia, múltiplas infiltrações prévias de corticóide, cooperação e aderência ao tratamento, ganhos secundários, etc. 2 3

Em resumo, fatores pré-operatórios que indicam baixa ou não possibilidade de reparo funcional são tamanho da lesão maior que 5 cm, substituição gordurosa do ventre acometido >50% (III ou mais de Goutallier), atrofia muscular >50% do ventre do músculo supraespinhal (sinal da tangente na ressonância magnética sequência sagital), retração do coto do tendão rompido ao nível da glenoide (Classificação de Patte III) e subluxação superior fixa da cabeça umeral, com altura acrômio umeral < 6mm (Classificação Hamada 2 ou mais). Esses paciente são considerados portadores de lesões irreparáveis e as opções de tratamento existentes são apresentadas na próxima seção. 2 3

## Tratamentos

- Tratamentos Clínicos
- Tratamentos Cirúrgicos que mantém a Articulação
- Tratamentos Cirúrgico que substituem a articulação

## Tratamento Clínico

O tratamento não cirúrgico é indicado INICIALMENTE para todos os pacientes sintomáticos, e mantido para os pacientes com baixa demanda funcional, e com comorbidades que contraindiquem o tratamento cirúrgico. Ele engloba reabilitação fisioterápica focada em medidas física anti-inflamatórias e analgésicas, reforço do remanescente do manguito, reforço da musculatura paraescapular e deltóide. Medicamentos anti-inflamatório não esteróides assim como infiltrações com corticóide devem ser utilizados com parcimônia. Não há evidência científica que justifique a utlização de injeção de ácido hialurônico ou PRP. 2

## Debridamento, Acromioplastia, Tenotomia com ou sem Tenodese

Todas essas modalidades de tratamento, juntas ou isoladamente, resultaram em melhora clínica imediata dos pacientes em torno de 95%, com deterioração progressiva da dor e função. Walch et al. 4 apresentaram o primeiro relato sistemático multicêntrico envolvendo 283 pacientes, com média de idade de 64,3 anos e seguimento médio de 57 meses. Observaram que o ganho inicial foi muito satisfatório em 93% dos pacientes, entretanto houve declínio da satisfação e função nos anos subsequentes, com consequente perda de rotação externa, atrofia e fraqueza, além de progressão da degeneração da cartilagem. Notaram também que a tenotomia tem efeito adverso em pacientes com distância acrômio-umeral >7mm, e que o acometimento do redondo menor tem valor prognóstico ruim.

Pander et al. 5 avaliaram retrospectivamente 39 pacientes, com média de idade de 75,6 anos, submetidos a debridamento de lesões irreparáveis, com ou sem tenotomia, e seguimento médio de 6,5 anos. Apesar de alguma fragilidade, por exemplo de não incluir os pacientes que necessitaram procedimento cirúrgico adicional devido a desfecho clínico insatisfatório, observou que é possível, na população idosa com baixa demanda funcional, obter resultados favoráveis.

Boileau et al. 6 observaram que a tenotomia do bíceps promoveu a ascensão da cabeça de 1,1mm em média, porém não é fator de risco para evolução para artropatia do manguito rotador. E sugeriram que a pseudoparalisia, assim como artropatia do manguito rotador, são contraindicações para esse tipo de procedimento.

Checchia et al. 7 também demonstraram que em 12 pacientes submetidos à tenotomia, com uma média de seguimento de 26 meses, houve uma melhora da satisfação, da amplitude de movimento 30° da elevação, 1,7° da rotação lateral, e de dois níveis vertebrais da rotação interna com apenas um caso de deformidade característica de Popeye. 7

Almeida et al mostraram que apenas 35,1% dos pacientes submetidos a tenotomia apresentavam queixas relacionadas com a deformidade do bíceps. E que idade, lado acometido e biótipo não apresentaram correlação significativa com queixas de deformidade, Por outro lado, sexo masculino, lado dominante, dobra cutânea abdominal <23,2 e triciptal< 14,5, e Índice de Massa Corporal (IMC) < 30 são fatores de risco para queixa de deformidade. 8

Em resumo, o debridamento, acromiolastia e tenotomia do bíceps, associada ou não a sua tenodese, devem ser reservados para o paciente idoso com baixa demanda funcional. Apresentam como vantagens ser uma técnica rápida, simples e com poucas complicações, quando comparadas com outras técnicas cirúrgicas. 35,1% dos pacientes submetidos à tenotomia queixam-se da deformidade residual (sinal do Popeye). Esses procedimentos juntos ou isolados apresentam melhora clínica imediata em 93%, que se deteriora com o tempo. O acometimento do tendão do músculo redondo menor, a subluxação superior estática com distância acrômio umeral< 6mm, degeneração cartilaginosas e pseudoparalisia são contra indicações.

## Reparo Parcial e Convergência Das Margens

Burkhart foi o primeiro autor a apresentar o conceito de lesão funcional do manguito rotador e reparo parcial. O que implica que os “tirantes" ou bordas das lesões, podem manter o manguito funcional a despeito da existência de uma lesão central, desde que essas forças residuais sejam equilibradas/compensadas, também conhecidas como "forças pares". Esse é o raciocínio lógico por detrás do reparo parcial, que é criar uma momento de força equilibrado no ombro. Muitas vezes, esse cenário é o de reparo de uma lesão aguda sobre crônica, quando a porção crônica da lesão é irreparável devido à má qualidade do tendão e grande retração, e a aguda é reparável. Quando a aguda cicatriza, é possível voltar ao estado de equilíbrio prévio ao traumatismo, com o manguito funcional devido às restauração das forças pares. Apesar de pequena, sua amostra inicial de 14 pacientes, seguimento médio de 20 meses, com média de idade de 56 anos, apresentou importante ganho na amplitude de movimento, força e satisfação dos pacientes. 9

Alem do reparo parcial, outro recurso cirúrgico para se incrementar biomecanicamente o reparo das lesões extensas, é a convergência das bordas. Quando, após o reparo parcial das margens anterior e posterior do manguito chegam com tensão aceitável ao “footprint" e parte central não, geralmente nas lesões em “V" ou “U”, a borda anterior e posterior são aproximadas e suturadas entre si, látero-lateralmente. Isso resulta em diminuição importante da força de arrancamento do manguito (força seis vezes menor), o que minimiza a falha da fixação e melhora dos resultados clínicos, que se mantêm estáveis no médio prazo. 10 11

Conclui-se que a indicação de reparo parcial, com ou sem convergência das margens é adequada para lesões extensas agudas sobre crônica (casos traumáticos), com parte do tendão subescapular preservado. Os melhores resultados são obtidos quando o tendões do subescapular e infraespinhal são reparados, restaurando o equilíbrio de forças, e tornando o manguito funcional, mesmo com a persistência de lesão em sua parte central. Observam-se bons e excelentes resultados em 67% dos pacientes no médio prazo. Resultado esse melhor que o debridamento isolado. 8 9 11

## Balão Subacromial

O balão subacromial/espaçador é um dispositivo inflável feito de Poli DL Lactídeo e E-Coprolactona que se degrada em 12 meses. O procedimento é simples e envolve a colocação desse dispositivo entre a cabeça umeral e o acrômio, que pode ser inserido por artroscopia ou de forma percutânea. Ele objetiva restaurar o arco de movimento indolor do ombro, na vigência de uma lesão irreparável, tanto por diminuir o atrito subacromial, como melhorando a alavanca do deltoide abaixando a cabeça umeral.

Senekovic foi um dos primeiros a demonstrar bons resultados com essa técnica. Ele mostrou que em 24 pacientes, com cinco anos de seguimento, ou seja, mesmo após a completa reabsorção do balão, 84,6% apresentaram melhora e apenas 10% apresentaram piora. O procedimento é considerado simples e a média de tempo para sua colocação é de 4,3 minutos. 12

A literatura indica que há melhora da dor e incapacidade funcional entre 46% e 84,6% dos casos e que a taxa de complicação é de 16,7%, sendo as mais frequentes: migração anterior do balão, déficit transitório do nervo cutâneo lateral do antebraço e infecção. A lesão irreparável do subescapular é contra-indicação devido ao risco de extravasamento anterior. 13

Sua indicação é em pacientes que apresentem lesão irreparável com integridade do subescapular e redondo menor, sem artrose e preferencialmente baixa demanda. Outra indicação menos clássica é para proteger a sutura de uma lesão do manguito ( Fig. 1 ). 12 13

**Fig. 1:** Desenho esquemático do balão subacromial.

## Reconstrução da Cápsula Superior (RCS)

Mihata et al. 14 desenvolveram o conceito de implantar a fáscia lata na glenoide e no tubérculo maior, para estabilizar a cabeça do úmero e impedir sua ascensão superior, características da lesão irreparável, restaurando o centro de rotação da cabeça do úmero. Essa capacidade foi testada "in vitro” em múltiplos trabalhos biomecânicos e reproduzida clinicamente desde então. 14 A fixação da cabeça umeral à glenoide, com o enxerto da fáscia lata, apresenta melhor resistência que a interposição do coto do tendão com outros enxertos. Os tecidos mais frequentemente interpostos são enxerto próprio da fásica lata (autólogo ou homólogo), enxerto dérmico acelular ou o tendão do bíceps (cabo longo). O ideal é suturar as bordas remanescentes do manguito ao enxerto (reparo parcial). 15

Os desfechos clínicos na literatura são diversos porque há muita heterogeneidade entres os procedimentos cirúrgicos: número de camadas da fáscia lata, tipos de enxertos (fácil lata, bíceps, etc.), método de local da fixação e experiência do cirurgião). Enquanto alguns autores não apresentam resultados consistentemente bons, Mihata descreveu o resultado em 31 ombros, de 23 pacientes, com média de idade de 65,1 anos, com melhora expressiva de 64° elevação anterior, 14° de rotação externa e 2 níveis vertebrais de rotação interna. 14 15 16

Denard et al. 17 usando enxerto dérmico relataram 18,6% de revisões cirúrgicas em 59 pacientes. E apesar de cicatrização completa em apenas 45% dos pacientes, 74,6% dos pacientes foram considerados bem sucedidos.

Com base nas informações precedentes, a indicação de RCS é para pacientes com lesões irreparáveis do manguito, sem degeneração cartilaginosa, refratários ao tratamento clínico, com subescapular íntegro ou reparável e Hamada 1 ou 2.

## Transferência Tendinosa

A indicação clássica para transferência tendinosa é o paciente jovem, em que sua idade seja uma contra indicação relativa para a artroplastia reversa, que não apresente degeneração cartilaginosa, e seja portador de lesão irreparável do manguito rotador. A transferência tendinosa pode apresentar resultados funcionais satisfatórios e duradouros nesse subconjunto de pacientes. Atualmente a maioria das transposições podem ser realizadas assistidas por artroscopia. Como regra geral, o músculo transposto fornece força em um nível menor que o músculo nativo, em condições fisiológicas. 18 19

Quando considerar a transposição tendinosa, há importantes princípios serem seguidos:

1 - O músculo transferido deve ser considerado dispensável, sem prejudicar a função do membro doador.
2 - O músculo doador e receptor devem ter excursão e tensão similares.
3 - O vetor de tensão dos músculos doador e receptor devem ser similares
4 - O músculo transferido deve substituir uma função do músculo receptor 18 19

## Lesões Póstero-Superiores

Historicamente, o latíssimo do dorso tem sido o músculo de escolha para essa transferência, entretanto, recentemente o trapézio inferior tem crescido em popularidade em virtude da facilidade, desfecho clínico e aderência aos princípios de transferência.

## Latíssimo do Dorso

É um músculo muito largo que tem como função a adução, extensão e rotação medial do braço. Origina-se a partir da fáscia toracolombar, processos espinhosos de T2 a L5 e face dorsal do sacro e crista ilíaca e também por meio de fascículos musculares provenientes de 3 ou 4 costelas inferiores, interdigitando-se com o oblíquo externo do abdome. Sua inserção é formada por um tendão terminal de 7 a 10 centímetros de comprimento por 0,5 a 1,5 centímetros de largura, após a torção axial de 180° no fundo do canal bicipital do úmero, entre os músculos peitoral maior anteriormente e o redondo maior posteriormente. Sua inervação motora se dá pelo nervo toracodorsal, ramo da corda posterior do plexo braquial. A sua vascularização primária é realizada pela artéria toracodorsal, que, juntamente com a artéria circunflexa da escápula, são ramos terminais da artéria subescapular. Do ponto de vista anatômico, duas estruturas estão em risco nas transferências desse músculo, o nervo axilar que está próximo à sua inserção e fica no trajeto da transposição, e o nervo radial que se localiza medial à sua inserção.

A seleção cuidadosa do paciente é crucial para obtenção de resultados satisfatórios e duradouros. Indica-se a transposição nas lesões extensas em pacientes jovens, ativos, sem degeneração cartilaginosa. São fatores de risco de mau prognóstico, portanto contra-indicações, lesões irreparáveis do tendão do subescapular, limitação da movimentação passiva, pseudoparalisia, artrose, Hamada 3 ou maior, e infiltração gordurosa do redondo menor. 18 19

Gerber foi quem popularizou a técnica e apresentou ótimos resultados no longo prazo, com 74% de viabilidade da transferência e bons e excelentes resultados no seguimento de 10 anos. O SSV aumentou de 29% para 70%, aumento significativo da amplitude de movimento e da força, com falha de 10% dos casos. 20

O local da inserção é fonte de controvérsia, acredita se que quando mais anterior (inserção do supraespinhal), aumente o efeito tenodese e quando mais posterior (inserção do infraespinhal) produza melhores momento de força para abdução e rotação externa. 18

A transposição do redondo maior associada ao latíssimo do dorso não incrementou de maneira significativa o desfecho clínico, dessa forma não é recomendada.

Atualmente com o aprimoramento técnico e material, a transposição totalmente artroscópica ou assistida apresenta os mesmos resultados clínicos e complicações. 18

## Lesões Anterossuperiores

## Peitoral Maior

O músculo peitoral maior aduz, flete e roda o úmero medial. Ele possui duas porções: a clavicular e esternal. A clavicular se origina do aspecto medial da clavícula e a esternal da porção superior do esterno, e da 2a a 4a costelas. Elas se inserem na parte lateral do sulco intertubercular. A irrigação provém do ramo peitoral do tronco tóraco acromial e a inervação dos nervos peitoral medial e lateral.

A sua transferência é a mais realizada para as lesões Antero superiores, para substituir as lesões irreparáveis do tendão do subescapular. Inicialmente descritas por Wirth e Rockwood, era transposto superficialmente ao tendão conjunto. Posteriormente a técnica foi revisada por Resch que o transpôs profundamente ao tendão conjunto, o que se mostrou mecanicamente mais eficiente. Atenção especial deve ser focada no nervo musculocutâneo, quando da transposição profunda ao tendão conjunto.

Os resultados clínicos são previsíveis para alívio da dor e heterogêneos com relação ao ganho funcional (ADM e força). Os resultados são melhores quando o tendão do músculo supraespinhal está preservado e cabeça centrada. Ao contrário, casos em que a cabeça umeral se encontra subluxada, apresentam piores resultados funcionais. 18

Uma explicação teórica para não reprodutibilidade do ganho funcional na transferência do peitoral maior é que em determinadas posições de abdução, o vetor de força do peitoral é ortogonal em relação ao vetor do subescapular, provocando mesmo a subluxação anterior do úmero. 18 19

## Latíssimo do Dorso

O latíssimo do dorso é acessado na fossa axilar e inserido, com ou sem o redondo maior, no tubérculo menor, quer seja aberto ou por via artroscopica. Essa técnica apresenta menor probabilidade de lesão iatrogênica de nervo e reproduz melhor a linha de tração do subescapular. 15 16

## Prótese Reversa

A indicação “padrão ouro" para a artroplastia reversa é a artropatia do manguito rotador (AMR), pois em um único procedimento solucionam-se os dois problemas: a degeneração articular e a insuficiência do manguito rotador decorrente de lesão irreparável, essa última de difícil solução com outras modalidades de cirurgia.

Enquanto na AMR está bem estabelecida a indicação da prótese reversa, na lesão irreparável com ausência de artrose, essa indicação não é tão clara e óbvia. A indicação deve ser baseada em critérios muito individualizados. 21

O candidato ideal deve ter idade maior que 65, dor e pseudoparalisia em um cenário de lesão irreparável, apresentar elevação anterior menor que 90°. Adicionalmente, pacientes idosos com outros fatores de mal prognóstico como tabagismo e diabetes também podem ser elegíveis para a artroplastia reversa, assim como pacientes que mesmo sem artrose, apresentem escape/instabilidade da cabeça do úmero. A literatura mostra que paciente submetidos artroplastia reversa em idade < 65 anos apresentam piores resultados clínicos e maiores taxas de complicação. 22

Contraindicações relativas são pacientes menores que 65 anos, disfunção neurológica do membro superior e pacientes com função “quase" normal com elevação maior que 90° e/ou resultado do questionário funcional "Simple Shoulder Test (SST)” > = 7, o que denota função satisfatória. 22 Até recentemente, a disfunção do músculo deltoide também era considerada contra indicação, entretanto Elhassan et al. demonstraram bons resultados nas artroplastias reversas associadas à transposição do peitoral em pacientes que possuíam paralisia do deltoide. 23

Frankle et al. 24 avaliaram 60 pacientes com média de seguimento de 33 meses, e mostraram que houve melhora significante de elevação anterior (55° para 105°), abdução (41° para 102°), rotação lateral (12° para 41°) e EVA escala visual analógica de dor (6,2 para 2,2). Em relação às complicações observaram 17% de “notching” escapular e 12% de revisão devido a falha da plataforma da glenóide. Esses resultados foram reproduzidos em múltiplos trabalhos por muitos cirurgiões. Trabalhos mais recentes como de Groh e Groh mostraram menores taxas de complicações (7%) e reoperações (5,3%). 25

Possivelmente a maior preocupação quando da colocação de uma prótese reversa seja a longevidade da prótese, principalmente nos pacientes mais jovens. A literatura mostra resultados entre 91% e 95% de sobrevida da prótese em 10 anos. E ao contrário de artroplastia para outras afecções, os casos de AMR e lesões irreparáveis apresentam menor deterioração funcional após o quinto ano de cirurgia ( Fig. 3 ). 26

**Fig. 3:** Fluxograma para o tratamento das lesões irreparáveis do manguito.

## Considerações Finais

O tratamento da lesão irreparável do manguito em paciente idoso, com baixa demanda funcional e artrose (artropatia do manguito rotador), apresenta resultado previsivelmente bom com a indicação de prótese reversa. Porém, há um subgrupo grande de pacientes jovens e ativos que apresentam critérios de irreparabilidade. Nesse subgrupo de pacientes, apesar do enorme avanço das modalidades cirúrgicas de tratamento nos últimos anos, desde o reparo parcial, passando pelo uso de balão subacromial, reconstrução da cápsula superior e transferências tendinosas, os resultados são menos previsíveis, em que pese a particularidade de cada paciente como personalidade da lesão (Anterossuperior, pósterossuperior, etc.), demanda funcional, aderência ao tratamento, comorbidades e, PRINCIPALMENTE, a experiência do médico assistente com as respectivas técnicas. 26

Na literatura, a maioria dos estudos apresentam nível de evidência III e IV, e em virtude da falta de estudos comparativos adequados, não há consenso em relação à superioridade de uma dessas técnicas sobre a as outras. Dessa forma, o tratamento deve ser individualizado, ponderando-se vantagens e desvantagens de cada modalidade para o respectivo paciente.

Em resumo, considerando-se a melhor evidência disponível, o **reparo parcial** é indicado em lesões aguda sobre crônica, dado o caráter reparável da “parte" aguda da lesão, e em pacientes sem artrose e de baixa demanda funcional. Apresentando resultados semelhantes à métodos mais invasivos. O **balão subacromial** é um método novo, sem aprovação de muitas agências reguladora, que tem indicação em pacientes sem artrose, com ADM preservada, subescapular íntegro e necessita de estudos mais robustos para confirmar sua indicação. A **reconstrução da cápsula superior** apresenta excelentes resultados nas mãos de cirurgiões experientes nessa técnica, pode ser utilizada em casos de pseudoparalisia, mas apresenta resultados frustrantes em caso de lesões irreparáveis do subescapular. As **transferências tendinosas do grande dorsal e trapézio inferior** para lesões pósterossuperiores, e do peitoral maior ou grande dorsal para as lesões anterossuperiores são boas indicações para pacientes jovens, com importante demanda funcional e sem artrose. Por fim, a prótese reversa é excelente indicação para paciente idosos com baixa demanda funcional, com ou sem artrose. Em pacientes menores de 60 anos, altos escores funcionais, grande demanda funcional, e/ou déficit neurológico os desfechos clínicos são piores.
